# Effect of sacubitril/valsartan on the occurrence of cardiac arrhythmias and the risk of sudden cardiac death in heart failure: A meta-analysis of randomized controlled trials

**DOI:** 10.3389/fcvm.2022.943377

**Published:** 2022-09-06

**Authors:** Xue-Hui Liu, Guan-Ling Wang, Qiang Xu, Lei Zhang, Hong-Jun Liu

**Affiliations:** ^1^Department of Cardiology, Yichang Hospital of Traditional Chinese Medicine, Yichang, China; ^2^Traditional Chinese Medicine Hospital of China Three Gorges University, China Three Gorges University, Yichang, China; ^3^Yichang Wujia Hospital, Yichang, China

**Keywords:** sacubitril/valsartan, cardiac arrhythmia, sudden cardiac death, heart failure, meta-analysis

## Abstract

**Background:**

Sacubitril/valsartan therapy reduced the risks of death and of hospitalization for heart failure (HF). HF and cardiac arrhythmias have shared physiological mechanisems. Therefore, sacubitril/valsartan may exhibit anti-arrhythmic properties in HF. The purpose of this study was to evaluate the effect of sacubitril/valsartan on the occurrence of cardiac arrhythmias and the risk of sudden cardiac death (SCD) in HF.

**Methods:**

This meta-analysis was performed according to PRISMA guidelines. We searched PubMed and Embase (from inception up to 6 February 2022) to identify randomized control trials (RCTs) on the effect of sacubitril/valsartan on the occurrence of cardiac arrhythmias and the risk of SCD in HF. Primary outcomes were the occurrence of atrial arrhythmias, ventricular arrhythmias, and SCD. Risk ratios (RRs) with 95% confidence intervals (CIs) were pooled using a random-effects model for meta-analysis.

**Results:**

We included 9 RCTs (published between 2012 and 2021) with 18,500 patients (9,244 sacubitril/valsartan vs. 9,256 active control). Enalapril and valsartan were used as active control in six and two studies, respectively. Follow-up ranged from 2 to 35 months. The cumulative occurrence of events was 76, 13, and 48 per 1,000 patient-years for atrial arrhythmias, ventricular arrhythmias and SCD, respectively. There was no significant association between sacubitril/valsartan therapy and the occurrence of atrial arrhythmias (RR 1.06; 95% CI: 0.97–1.17; *P* = 0.19) and ventricular arrhythmias (RR 0.86; 95% CI 0.68–1.10; *P* = 0.24). However, sacubitril/valsartan therapy significantly reduced the risk of SCD (RR 0.79; 95% CI 0.70–0.90; *P* = 0.03) compared with control.

**Conclusion:**

No association between sacubitril/valsartan therapy and the occurrence of atrial and ventricular arrhythmias was found, but sacubitril/valsartan therapy significantly reduced the risk of SCD.

## Introduction

Heart failure (HF) is associated with substantial morbidity and mortality. Cardiac arrhythmias are common in HF, and HF predisposes cardiac arrhythmias and vice versa (1). Cardiac arrhythmias, including atrial arrhythmias and ventricular arrhythmias, are important causes of adverse outcomes in HF patients (1–3). Sudden cardiac death (SCD) is also a major cause of mortality among HF patients and is commonly related to ventricular arrhythmias, particularly ventricular tachycardia (VT) and ventricular fibrillation (VF) (4). The management of cardiac arrhythmias in HF depends on the type and etiology of arrhythmia, the severity of HF, and the range from medical therapy to cardiac implantable electronic devices (CIEDs) (2, 4). Previous studies suggest that drugs blocking the rennin-angiotensin-aldosterone system (RAAS) and natriuretic peptide (NP) system have various beneficial effects on arrhythmia mechanisms (4, 5).

Sacubitril/valsartan, an angiotensin receptor-neprilysin inhibitor (ARNI), has been shown to reduce the risk of cardiovascular death or HF hospitalization in patients with HF compared with enalapril (6). The advantages of sacubitril/valsartan are likely to result from reduced cardiac remodeling, improved left ventricular ejection fraction (LVEF), and increased NP availability (7). Therefore, sacubitril/valsartan may exhibit anti-arrhythmic properties and modulate the risk of cardiac arrhythmias in HF.

Two recent meta-analyses on the similar topic have been published (8, 9). The meta-analysis by Fernandes et al. found that ARNI therapy was associated with a reduction in SCD and ventricular arrhythmias compared with control in HF with reduced ejection fraction (HFrEF) (8). However, the role of sacubitril/valsartan in HF with preserved ejection fraction (HFpEF) remains unclear. Another meta-analysis by Liu et al. found that sacubitril/valsartan was similar to control in preventing the occurrence of atrial fibrillation (AF) in HF (9). The effect of sacubitril/valsartan on the risks of ventricular arrhythmias and SCD was not evaluated. Recently, several randomized controlled trials (RCTs) (10–12) involving more evidence have been published. Thus, a comprehensive evaluation of the effect of sacubitril/valsartan on this topic is needed. The purpose of this meta-analysis was to evaluate the effect of sacubitril/valsartan on the occurrence of cardiac arrhythmias and risk of SCD in patients with HF.

## Methods

This meta-analysis was conducted according to the Preferred Reporting Items for Systematic Reviews and Meta-analyses (PRISMA) guidelines (13).

### Search strategy

PubMed and Embase were searched from inception up to 6 February 2022. Search terms included “sacubitril,” “sacubitril/valsartan,” “LCZ696,” “neprilysin,” and “randomized controlled trial.” No language restriction was applied. References of included trials and previous reviews were checked for potentially eligible trials.

### Study selection and eligibility criteria

Two authors independently reviewed the titles and abstracts of all articles initially identified, according to the inclusion criteria. Disagreements were resolved by discussion.

Studies were included if they met the following criteria: (1) randomized controlled trials; (2) adult patients older than 18 years; (3) presented of a control group (either placebo or active controlled); and (4) reported the outcomes of interest as an endpoint or adverse events (AEs). The outcomes included the occurrence of atrial arrhythmias [AF, atrial flutter (AFL), and atrial tachycardia (AT)], ventricular arrhythmias [VF, ventricular flutter (VFL), and VT], and SCD (sudden cardiac death, sudden death, and cardiac arrest).

### Data extraction

Two authors independently extracted the following data from the included trials: first author, publication year, ClinicalTrials.gov unique identifier, study characteristics, and outcomes of interest. When multiple publications of the same trial were found, data from the most complete dataset were extracted for analysis. If no outcomes of interest were reported in the manuscript, we searched the supplementary material and the adverse event of the trial on ClinicalTrials.gov. Disagreements were resolved by discussion.

### Assessment of risk of bias

The risk of bias of included trials was assessed by using the Reviews Manager 5.4.1, which included the following sections: selection bias, performance bias, detection bias, attrition bias, reporting bias, and other bias. Trial with one or more key domains at high risk of bias was judged to high risk of bias; trial with all key domains at low risk of bias was judged to low risk of bias; otherwise it was judged to unclear risk of bias (14). The results were presented as a risk of bias graph and a risk of bias summary figure.

### Statistical analysis

Risk ratios (RRs) with 95% confidence intervals (CIs) were used to calculate the pooled effects. Meta-analyses were conducted using a random-effects model regardless of heterogeneity. Statistical heterogeneity across studies was assessed by the I^2^ statistic (15). An I^2^ value greater than 50% indicates significant heterogeneity. Publication bias was not performed because the number of included trials was too small (<10) to detect an asymmetric funnel. Subgroup analyses were pre-specified according to the type of HF (HFrEF vs. HFpEF), control agent used, follow-up duration (<1 year vs. >1 year). A two-sided *P*-value < 0.05 was considered statistically significant. All analyses were performed using Review Manager Software (RevMan version 5.4; The Nordic Cochrane Centre, Cochrane Collaboration).

## Results

### Study search

**FIGURE 1:**
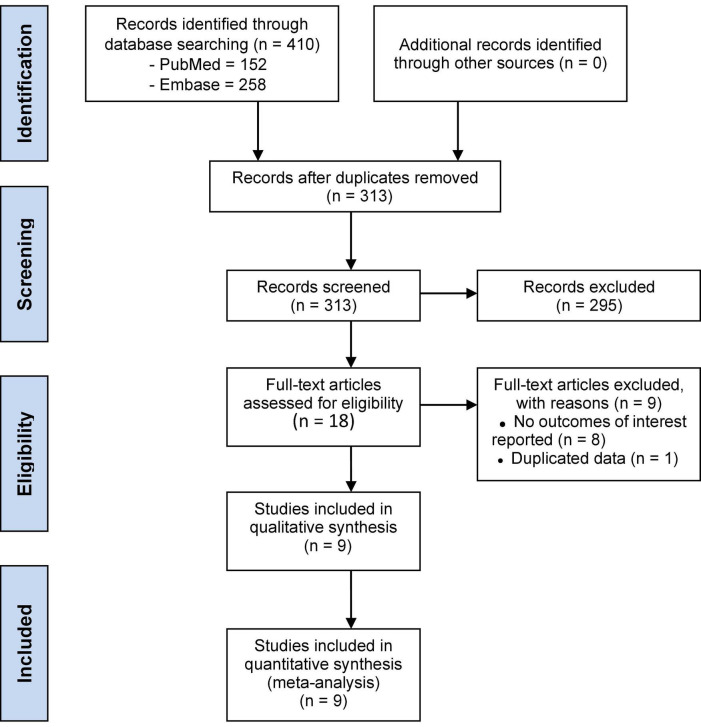
Flow diagram of search strategy.

A total of 410 articles were initially identified, of which 9 RCTs (6, 10–12, 16–20) were included in the meta-analysis, comprising a total of 18,500 patients, of whom 9,244 were in the sacubitril/valsartan group and 9,256 in the control group. The search strategy is presented in Figure 1.

### Characteristics of eligible studies

The baseline characteristics of the included studies are summarized in Table 1. All trials were randomized, double-blind, and active-control. The trials were published between 2012 and 2021. Among the included trials, 6 trials (6, 10, 12, 17, 19, 20) used enalapril as a comparator, 2 (16, 18) used valsartan as a comparator, and 1 (11) used individualized medical therapy (IMT) as a comparator. Of included nine trials, 6 trials (6, 10, 12, 17, 19, 20), including 1 (19) enrolled patients with acute decompensated HF, enrolled patients with HFrEF the others (11, 16, 18) enrolled patients with HFpEF. The sample size ranged from 201 to 8,432. The mean age ranged from 62 to 72.8 years, and the percentage of women ranged from 13.9 to 56.5%, with a mean follow-up duration between 2 and 35 months. All included trials did not describe the definition of cardiac arrhythmias and the methods used to document cardiac arrhythmias. All included trials were funded by industry.

**TABLE 1:** Baseline characteristics of the included trials.

	PARAMOUNT, 2012		PARADIGM-HF, 2014		PARAGON-HF, 2019		EVALUATE-HF, 2019		PIONEER-HF, 2019		OUTSTEP-HF, 2019	
						
	Sac/Val (*n* = 149)	Valsartan (*n* = 152)	Sac/Val (*n* = 4203)	Enalapril (*n* = 4229)	Sac/Val (*n* = 2419)	Valsartan (*n* = 2,402)	Sac/Val (*n* = 231)	Enalapril (*n* = 233)	Sac/Val (*n* = 439)	Enalapril (*n* = 436)	Sac/Val (*n* = 309)	Enalapril (*n* = 310)
NCT	00887588		01035255		01920711		02874794		02554890		02900378	
Follow-up duration	36 weeks		27 months		35 months		12 weeks		8 weeks		12 weeks	
Does of Sac/Val	Started with 50 mg bid for 1–2 weeks, then uptitrated to 100 mg bid for 1–2 weeks, and thereafter, uptitrated to 200 mg bid.		200 mg bid during double blind treatment period		Target dose of Sac/Val during the double blind period was 200 mg bid		Started with 50 mg bid, and titrated every 2 weeks to 200 mg bid		Target dose of Sac/Val was 200 mg bid		Started with 50 mg bid or 100 mg bid, then uptitrated to a target dose of 200 mg bid	
Age (years)	70.9(9.4)	71.2(8.9)	63.8(11.5)	63.8(11.3)	72.7(8.3)	72.8(8.5)	67.8 (9.8)	66.7 (8.5)	61 median	63	67.2(11.0)	66.6(10.5)
Women	57%	56%	21.0%	22.6%	51.6%	51.8%	26%	21%	25.7%	30.2%	23.0%	19.7%
BMI	30.1(5.5)	29.8(6.1)	28.1(5.5)	28.2(5.5)	30.2(4.9)	30.3(5.1)	30.0(5.7)	30.1 (5.8)	30.5 med	30.0	29.3(4.7)	29.3(4.7)
Serum creatinine (mg/dl)	NA	NA	1.13 (0.3)	1.12 (0.3)	NA	NA	NA	NA	1.28	1.27	NA	NA
eGFR	67 (19)	64 (21)	NA	NA	63 (19)	62 (19)	70 (22)	69 (20)	58.4	58.9	NA	NA
NYHA functional class I II III IV	1% 81% 19% 0%	1% 78% 21% 0%	4.3% 71.6% 23.1% 0.8%	5.0% 69.3% 24.9% 0.6%	3% 77.5% 19.0% 0.3%	2.7% 77.0% 19.8% 0.5%	14% 66% 20% 0%	12% 69% 19% 0%	0.9% 22.7% 64.3% 8.9%	1.1% 27.7% 61.0% 8.2%	0% 52.1% 47.3% 0.6%	0% 52.3% 47.1% 0.6%
Hypertension	95%	92%	70.9%	70.5%	95.7%	95.4%	NA	NA	NA	NA	68.9%	65.5%
Diabetes	41%	35%	34.7%	34.6%	43.5%	42.5%	NA	NA	NA	NA	NA	NA
AF or AFL	40%	43%	AF 36.2	37.4%	32.2%	32.5%	NA	NA	NA	NA	47.6%	39.4%
MI	21%	20%	43.4%	43.1%	23.3%	21.9%	NA	NA	NA	NA	44.3%	46.8%
Stroke	NA	NA	8.5%	8.8%	11.1%	10.1%	NA	NA	NA	NA	7.1%	8.1%
Medical therapy at randomization β-blocker MRA Diuretic	79% 19% 100%	80% 23% 100%	93.1% 54.2% 80.3%	92.9% 57.0% 80.1%	79.9% 24.6% 95.3%	79.5% 27.1% 95.9%	85% 25% 56%	88% 25% 55%	59.5% 10.9% 59.5%	59.6% 9.1% 54.4%	90.6% 64.4% 77.7%	92.6% 69.4% 75.5%

	**ACTIVITY-HF, 2021**				**PARALLAX, 2021**				**PARALLEL-HF, 2021**			
			
	**Sac/Val** **(*n* = 103)**		**Enalapril** **(*n* = 98)**		**Sac/Val** **(*n* = 1280)**		**IMT** **(*n* = 1284)**		**Sac/Val** **(*n* = 111)**		**Enalapril** **(*n* = 112)**	

NCT	02768298				03066804				02468232			
Follow-up duration	12 weeks				24 weeks				33.9 months			
Does of Sac/Val	100 mg bid for 2 weeks followed by 200 mg bid for 10 weeks.				Target dose of Sac/Val was 200 mg bid				Started with 50 mg bid, then uptitrated to a target dose of 200 mg bid.			
Age (years)	66.1(10.8)		67.6(10.0)		72.9 (8.4)		72.4 (8.6)		69.0 (9.7)		66.7 (10.9)	
Women	16.5%		21.4%		50.2%		51.2%		13.5%		14.3%	
BMI (kg/m^2^)	29.2(4.6)		29.6(4.3)		30.6 (5.0)		30.5 (4.8)		23.8 (4.0)		25.1 (4.2)	
Serum creatinine (mg/dl)	NA		NA		NA		NA		NA		NA	
eGFR (ml/min/1.73 m^2^)	NA		NA		62.5 (20.2)		62.7 (19.6)		58.3 (17.6)		57.6 (14.7)	
NYHA functional class I II III IV	0% 0% 100% 0%		0% 1% 99.0% 0%		0.1% 67.0% 32.5% 0.4%		0.3% 68.2% 31.2% 0.3%		3.6% 91.0% 5.4% 0%		3.6% 92.9% 3.6% 0%	
Hypertension	NA		NA		96.9%		97.4%		64.0%		73.2%	
Diabetes	NA		NA		44.2%		45.8%		46.8%		46.4%	
AF or AFL	NA		NA		54.6%		53.9%		32.4%		35.7%	
MI	56.3%		55.1%		23.0%		23.8%		46.0%		41.1%	
Stroke	NA		NA		NA		NA		9.9%		8.9%	
Medical therapy at randomization β-blocker MRA Diuretic	92.2% 78.6% 76.7%		96.9% 75.5% 76.5%		83.7% 32.7% 99.8%		83.0% 30.5% 99.8%		94.6% 57.7% 82.0%		96.4% 61.6% 84.8%	

Data are mean (standard deviation); AF, atrial fibrillation; AFL, atrial flutter; BMI, body mass index; eGFR, estimated glomerular filtration rate; IMT, individualized medical therapy; MI, myocardial infarction; MRA, mineralocorticoid receptor antagonist; NA, data not available; NYHA, New York Heart Association; Sac/Val, sacubitril/valsartan.

### Assessment of risk of bias

**FIGURE 2:**
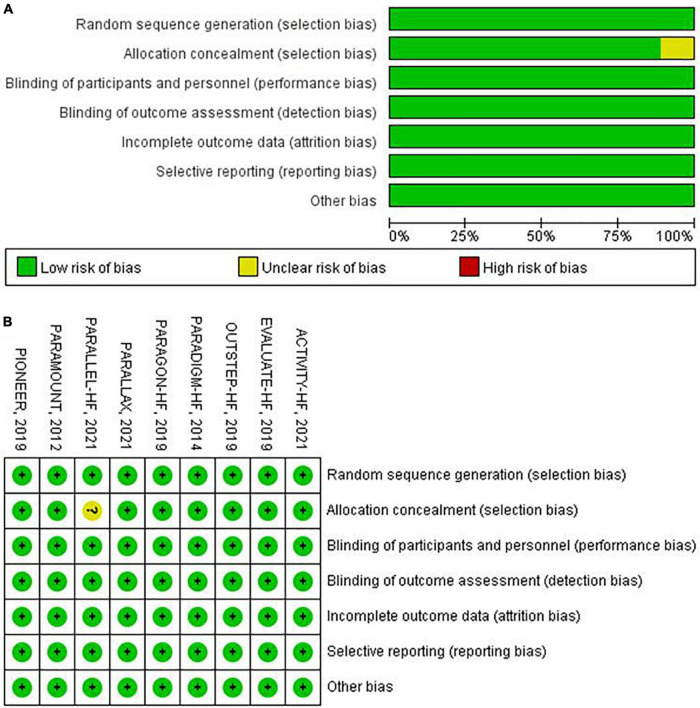
Assessment of risk of bias. **(A)** Risk of bias graph, **(B)** risk of bias summary.

Details of risk of bias assessment are summarized in Figure 2. Eight trials (6, 10, 11, 16–21) were judged to be at low risk of bias, and one trial (12) was judged to be at unclear risk of bias.

### Atrial arrhythmias

**FIGURE 3:**
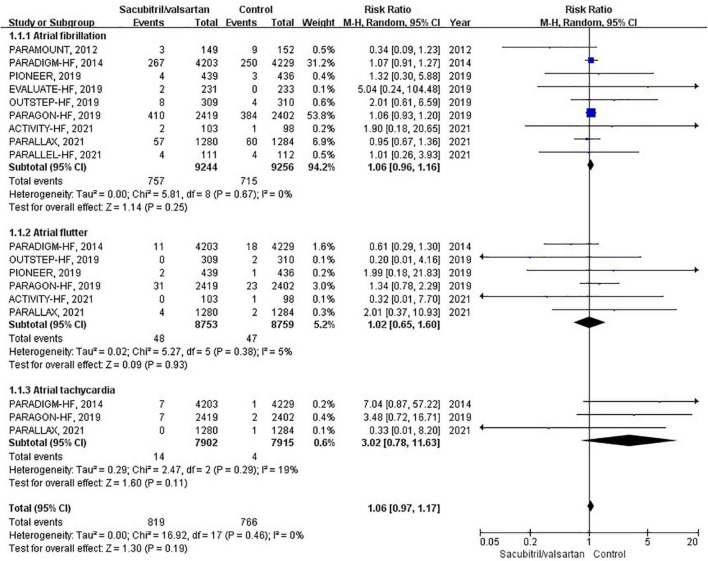
Forest plot comparing the occurrence of atrial arrhythmias between sacubitril/valsartan and control.

Atrial arrhythmias were reported in 9 RCTs (6, 10–12, 16–20), of which 6 compared sacubitril/valsartan with enalapril, 2 with valsartan, and 1 with IMT. During an average follow-up of 1.13 years, the cumulative occurrence of atrial arrhythmias was 78 per 1,000 patient-years in the sacubitril/valsartan group and 73 per 1,000 patient-years in the control group. The occurrence of atrial arrhythmias was not significantly different between the sacubitril/valsartan and control group (RR 1.06; 95% CI: 0.97–1.17; *P* = 0.19; Figure 3). There was no heterogeneity across the studies (I^2^ = 0%, *P* = 0.46). The pooled effects of 3 pre-specified components of atrial arrhythmias (AF, AFL, AT) were individually presented in Figure 3.

To assess the effect of sacubitril/valsartan therapy in HFrEF and HFpEF, we conducted corresponding subgroup analyses. However, neither HFrEF (RR 1.08; 95% CI: 0.92–1.26; *P* = 0.33) nor HFpEF (RR 1.00; 95% CI: 0.78–1.30; *P* = 0.98) showed a significant association.

In subgroup analysis based on comparator used, sacubitril/valsartan therapy was associated with no significant difference in the occurrence of atrial arrhythmias compared with enalapril (RR 1.08; 95% CI: 0.92–1.26; *P* = 0.33) or valsartan (RR 0.73; 95% CI: 0.25–2.16; *P* = 0.57).

Regarding the follow-up duration, we defined two subgroups, shorter duration (<1 years; RR 0.98; 95% CI: 0.73–1.33; *P* = 0.90) and longer duration (>1 years; RR 1.08; 95% CI: 0.98–1.19; *P* = 0.12), and neither affected the occurrence of atrial arrhythmias.

### Ventricular arrhythmias

**FIGURE 4:**
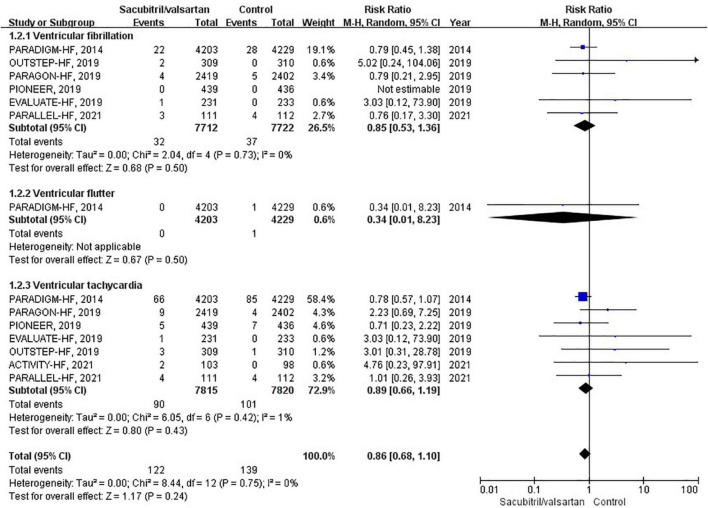
Forest plot comparing the occurrence of ventricular arrhythmias between sacubitril/valsartan and control.

Ventricular arrhythmias were reported in 7 RCTs (6, 10, 12, 17–20), of which 6 compared sacubitril/valsartan with enalapril, and 1 with valsartan. A total of 262 events of ventricular arrhythmias were reported as AEs. During an average follow-up of 1.27 years, the cumulative occurrence of ventricular arrhythmias was 12 per 1,000 patient-years in the sacubitril/valsartan group and 14 per 1,000 patient-years in the control group. The occurrence of ventricular arrhythmias was not significantly different between the sacubitril/valsartan and control group (RR 0.86; 95% CI 0.68–1.10; *P* = 0.24; Figure 4). There was no significant heterogeneity across studies (I^2^ = 0%, *P* = 0.75). The 3 pre-specified components of ventricular arrhythmias (VF, VFL, VT) were individually presented in Figure 4.

To assess the effect of sacubitril/valsartan therapy in HFrEF and HFpEF, we conducted corresponding subgroup analyses. However, neither HFrEF (RR 0.89; 95% CI: 0.60–1.33; *P* = 0.58) nor HFpEF (RR 1.43; 95% CI: 0.61–3.35; *P* = 0.40) showed a significant association.

In subgroup analysis based on comparator used, sacubitril/valsartan therapy was associated with no significant difference in the occurrence of ventricular arrhythmias compared with enalapril (RR 0.89; 95% CI: 0.60–1.33; *P* = 0.58) or valsartan (RR 1.43; 95% CI: 0.61–3.35; *P* = 0.40).

Regarding the follow-up duration, we defined two subgroups, shorter duration (<1 years; RR 1.91; 95% CI: 0.56–6.47; *P* = 0.30) and longer duration (>1 years; RR 0.83; 95% CI: 0.65–1.07; *P* = 0.16), and neither affected the occurrence of ventricular arrhythmias.

### Sudden cardiac death

**FIGURE 5:**
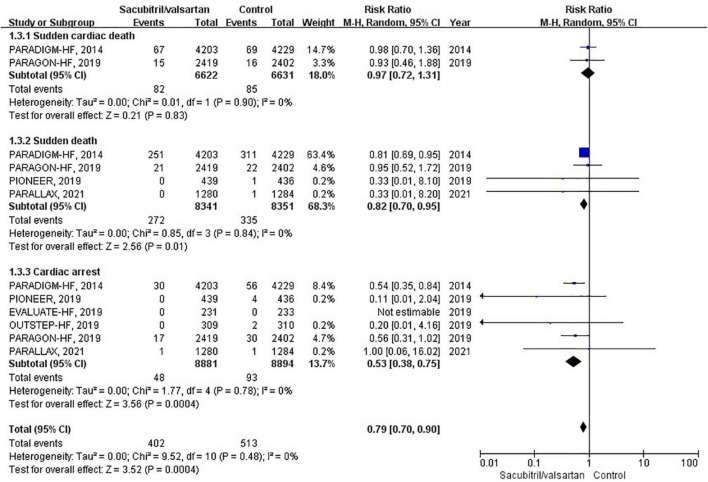
Forest plot comparing the risk of sudden cardiac death between sacubitril/valsartan and control.

Sudden cardiac death was reported in 6 RCTs (6, 11, 17–20). During an average follow-up of 1.06 years, the cumulative occurrence of SCD was 43 per 1,000 patient-years in the sacubitril/valsartan group and 54 per 1,000 patient-years in the control group. The overall analysis of the composite SCD outcome demonstrated a 21% reduction when compared with control (RR 0.79; 95% CI 0.70–0.90; *P* = 0.03; Figure 5). The 3 pre-specified components of SCD (sudden cardiac death, sudden death, and cardiac arrest) were individually presented in Figure 5.

## Discussion

To our knowledge, this is the largest and most comprehensive meta-analysis that evaluates the association between sacubitril/valsartan and the risk of arrhythmic events. Our meta-analysis found no association between sacubitril/valsartan therapy and the occurrence of atrial and ventricular arrhythmias. But it significantly reduced the risk of SCD in patients with HF.

Two previous meta-analyses on the similar topic have been published. One meta-analysis of six RCTs by Liu et al. showed no association between sacubitril/valsartan therapy and the occurrence of AF in patients with HF compared with control (9). In comparison, this meta-analysis added three latest published trials (10–12) and evaluated the effect of sacubitril/valsartan on the occurrence of ventricular arrhythmias and the risk of SCD in HF. Similar to previous meta-analysis, no significant association between sacubitril/valsartan and the occurrence of atrial arrhythmias, including AF, was observed. Another meta-analysis by Fernandes et al. concluded that ARNI therapy was associated with lower SCD events and ventricular arrhythmias compared with control in HFrEF (8). However, the finding was underpowered limited to the included observational studies. Observational studies are highly subject to selection bias. If only RCTs were included in their meta-analysis, there was no significant difference between groups regarding of the occurrence of ventricular arrhythmias. Besides, the association between sacubitril/valsartan and the risk of cardiac arrhythmias in HFpEF was not evaluated. In contrast with the previous ones, our meta-analysis is the latest and the most comprehensive.

**FIGURE 6:**
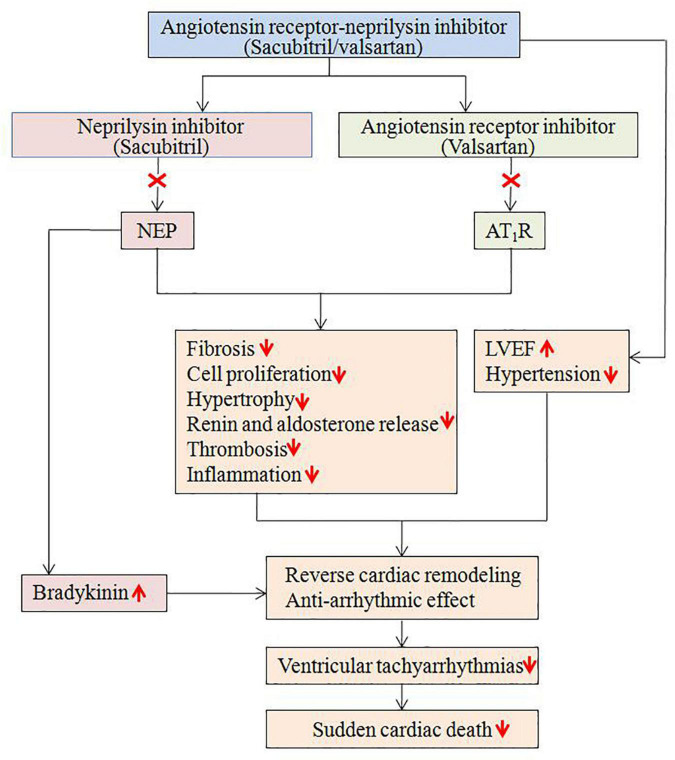
Potential mechanisms regarding the anti-arrhythmic effects of sacubitril/valsartan. AT1R, angiotensin II type1 receptor; LVEF, left ventricular ejection fraction; NEP, neprilysin.

It is well known that HF is associated with increased risk of cardiac arrhythmias and SCD, which is related to multiple potential mechanisms, including the RAAS and NP system (4, 5). The RAAS and NP system play important role in the development of structural and electrical remodeling (5), potentially explaining the occurrence of cardiac arrhythmias. Sacubitril/valsartan has been shown positive results on patients’ outcome, particularly in those with HF (22). In the PARAMOUNT study, sacubitril/valsartan therapy resulted in greater reduction in NT-proBNP at 12 weeks and greater reduction in left atrial size after 36 weeks compared with valsartan (16). In the PARADIGM-HF study, the further reduction of cardiovascular mortality, including SCD, observed in HFrEF received sacubitril/valsartan is likely due to a combined protective effect against death from HF and fatal ventricular arrhythmias (6, 23, 24). A retrospective study demonstrated sacubitril/valsartan therapy was associated with improvements in echocardiographic parameters, including LVEF, pulmonary atrial pressure and cardiac valvular insufficiency, in patients with HFrEF (25). To date, increasing evidence suggests that sacubitril/valsartan may have anti-arrhythmic properties, either by limiting pro-arrhythmic remodeling or through direct anti-arrhythmic effects on cardiomyocytes (Figure 6) (5, 26–28). Although these mechanisms of sacubitril/valsartan are potential contributors to the observed *in vivo* anti-arrhythmic effects, there is no conclusive mechanism regarding sacubitril/valsartan mediated cardiac arrhythmia suppression in patients.

Based on the results of our meta-analysis, sacubitril/valsartan therapy does not reduce the occurrence of cardiac arrhythmias in patients with HF. One possible explanation is that all included RCTs were active-control trials. The renin-angiotensin system inhibitions are associated with reduction in cardiac arrhythmias (2, 29–31), and the incremental benefit of sacubitril/valsartan therapy for this outcome may have been minimal. Another possible explanation is that all included RCTs were not designed to evaluate the effect of sacubitril/valsartan on cardiac arrhythmias and the actual occurrence of cardiac arrhythmias may have been underestimated since not all patients underwent continuous rhythm monitoring. In HFrEF patients with CIEDs, previous studies have suggested that sacubitril/valsartan could decrease atrial arrhythmia burden and reduce the recurrence of atrial arrhythmias in patients with non-permanent AF (32, 33). Diego et al. found that sacubitril/valsartan could decrease ventricular arrhythmias in HFrEF patients under continuous monitoring of ICD compared with angiotensin inhibition (34). Furthermore, appropriate ICD shocks were significantly reduced. However, a study presented that sacubitril/valsartan does not reduce the risk of ventricular arrhythmias in HFrEF patients over 12 months of follow-up (35). In addition, another retrospective study reported that male and previous episodes of ventricular arrhythmias could be associated with an increased risk of sustained ventricular arrhythmias after sacubitril/valsartan initiation (36). Overall, most studies suggest sacubitril/valsartan might reduce the risk of cardiac arrhythmias in HF patients.

Sudden cardiac death is the leading cause of mortality in HF (37). In most cases, SCD is triggered by ventricular arrhythmias (38). Implantable cardioverter defibrillator (ICD) and wearable cardioverter defibrillator (WCD) are recommended for the prevention of SCD in selected populations (39, 40). Our meta-analysis showed that there was a 21% reduction in the risk of SCD. A *post-hoc* analysis of PARADIGM-HF demonstrated that sacubitril/valsartan reduced SCD risk regardless of ICD use (HR 0.49; 95% CI 0.25–0.99) or eligibility criteria (HR 0.81; 95% CI 0.67–0.98) in HFrEF (23). Given this outstanding advantage, sacubitril/valsartan is recommended to reduce SCD in HFrEF (2). The possible explanation is that sacubitril/valsartan could lead to reverse cardiac remodeling and attenuation of myocardial fibrosis (17, 41), both of which may reduce the risk of ventricular arrhythmias.

Because of the potential adverse outcomes of HF patients who develop cardiac arrhythmias, an upstream therapy with sacubitril/valsartan may prevent or delay the development of cardiac arrhythmias. According to our meta-analysis, it is premature to recommend sacubitril/valsartan solely for the prevention of cardiac arrhythmias, but our findings raise the possibility of an added benefit in HF patients receiving ARNI therapy. For selected patients, WCD in addition to sacubitril/valsartan treatment of HF is a possible approach to bridge the time until improvement of LVEF.

There are several potential limitations to our meta-analysis. First, events of cardiac arrhythmias and SCD in the included RCTs were reported as adverse events, and not as pre-specified endpoints. Although the number of cardiac arrhythmias was coded by reported adverse events, it is difficult to exclude the fact that some of the patients had asymptomatic arrhythmias that converted spontaneously. Second, there were no standardized definition and routine monitoring for the cardiac arrhythmias in the included RCTs, which may lead to reporting bias. It is not known whether the sudden death and cardiac arrest represented death from an arrhythmia or from another mechanism. Third, cardiac arrhythmias were not described in terms of sustained or non-sustained, fast or slow. Forth, data have shown a possible different effects of sacubitril/valsartan according to the HF etiology and age (42, 43). Due to lack of data, the effects of sacubitril/valsartan on the risk of cardiac arrhythmias and SCD according to HF etiology and age remain unclear. Finally, no cardiac MRI was done to correlate arrhythmias with fibrosis. These limitations should be considered when interpreting our findings.

## Conclusion

No association between sacubitril/valsartan therapy and the occurrence of atrial and ventricular arrhythmias was found, but it significantly reduced the risk of SCD. On the basis of our findings, we suggest that future RCTs systematically detect cardiac arrhythmias with routine ambulatory monitoring and define them as primary endpoints.

## Data availability statement

The original contributions presented in this study are included in the article/supplementary material, further inquiries can be directed to the corresponding author.

## Author contributions

X-HL, G-LW, and QX conceived the study, participated in the design, data collection, and statistical analysis. X-HL drafted the manuscript. LZ participated in the design. H-JL conceived the study, participated in the design, and critical revision of the manuscript. All authors read and approved the final manuscript.
